# Does culture create craving? Evidence from the case of menstrual chocolate craving

**DOI:** 10.1371/journal.pone.0181445

**Published:** 2017-07-19

**Authors:** Julia M. Hormes, Martha A. Niemiec

**Affiliations:** University at Albany, State University of New York, Albany, New York, United States of America; Indiana University Bloomington, UNITED STATES

## Abstract

Craving is considered a key characteristic of diverse pathologies, but evidence suggests it may be a culture-bound construct. Almost 50% of American women crave chocolate specifically around the onset of menstruation. Research does not support popular accounts implicating physiological factors in menstrual chocolate craving etiology. We tested the novel hypothesis that greater menstrual craving prevalence in the U.S. is the product of internalized cultural norms. Women of diverse backgrounds (*n* = 275) reported on craving frequency and triggers and completed validated measures of acculturation. Foreign-born women were significantly less likely to endorse menstrual chocolate craving (17.3%), compared to women born to U.S.-born parents (32.7%, *p* = .03) and second generation immigrants (40.9%, *p* = .001). Second generation immigrant and foreign-born women endorsing menstrual chocolate craving reported significantly greater U.S. acculturation and lower identification with their native culture than non-menstrual cravers (all *p* < .001). Findings inform our understanding of food cravings, with important implications for the study of cravings in other domains.

## Introduction

Craving is now widely considered a key characteristic of diverse pathologies, including weight and eating disorders, substance and non-substance addictions, impulse control and obsessive compulsive disorders, and paraphilias [1–6]. The role of craving in relapse into addictive behaviors has been widely documented [7–10]. Craving is also thought to play a role in food addiction [11], and to act as a powerful trigger in the onset of binge eating episodes in patients with bulimia nervosa and binge eating disorder, and a barrier to the successful achievement and maintenance of weight loss [4,12,13]. The role of internal factors such as stress or negative affect in the onset of craving episodes is generally recognized [14]. Research has also increasingly acknowledged the importance of external context and situational cues in craving etiology [15]. This study sought to expand upon our understanding of the importance of contextual factors in the emergence of craving by examining the role of culture in a specific and well-documented instance of craving, namely cyclically occurring craving for chocolate.

Chocolate cravings are endorsed by over 90% of U.S. women [16]. In about half of these female cravers, chocolate craving frequency and intensity markedly increase specifically around the onset of menstruation [17]. This striking temporal pattern is the subject of ongoing speculation. Popular accounts regarding menstrual chocolate craving etiology implicate cyclically fluctuating hormones or ingredients in chocolate thought to either alleviate premenstrual nutritional deficits or remedy premenstrual symptoms via their pharmacological effects. Remarkably, in spite of continuing resistance against abandoning a largely biomedical view of menstrual craving, empirical evidence has consistently failed to support these hypotheses [18,19].

While general and menstrual chocolate cravings are common in the U.S., they are exceedingly rare in other parts of the world. For example, a mere 6% of Egyptian women endorse craving chocolate [20]. Only 28% of Spanish women experience menstrual cravings [16]. These geographic differences hint at a possible causal role of internalized cultural norms: in a society that emphasizes the “thin ideal” of female beauty, women may view menstruation as a socially sanctioned excuse to indulge in an otherwise “taboo” food, resulting in cyclic increases in reported craving frequency [16]. This study sought to test the surprising, yet increasingly compelling hypothesis that culture is causally implicated in menstrual chocolate craving etiology by assessing 1. chocolate craving prevalence in women of diverse cultural backgrounds and 2. associations between menstrual craving and U.S. acculturation.

## Materials and methods

The Institutional Review Board at the University at Albany reviewed and approved all methods. Participants were informed of the nature and purpose of the research and consented prior to completion of questionnaires.

### Procedures and participants

Data were collected as part of a larger survey study examining types and frequency of food cravings in individuals from diverse cultural backgrounds. Participants included in the present analyses were undergraduate women (*n* = 275) who received research participation credit in exchange for time spent in the laboratory. Recruitment materials specifically targeted individuals (a) born outside the U.S., (b) with parent(s) or primary guardian(s) born outside the U.S., (c) who primarily lived outside the U.S. until age 18 and/or (d) primarily spoke a language other than English while growing up.

### Measures

Participants completed a battery of self-report questionnaires while seated at individual computer stations to ensure privacy. They were asked to provide information on age, height and weight, birthplace, time spent in the U.S. and abroad, native language(s), and parents’ birthplace and current residence.

#### Chocolate craving

Participants reported on the presence of “any” (“Have you ever experienced a craving for chocolate?”–rated “yes or “no”) and “regular” chocolate cravings (“Do you crave chocolate regularly?”–rated “yes” or “no”), in a manner comparable to prior studies of craving [14,21] (see S1 Appendix). If participants provided a positive response to “yes”/”no” questions about temporal patterns (“If you experience any chocolate cravings, do you feel that they occur at any specific point in time (i.e. time of year, month, day, etc.)?”) and perceived triggers of chocolate cravings (“If you experience any chocolate cravings, do you feel that they are caused by any particular triggers?”), they were asked to provide additional details in open-ended questions (“If YES, please specify the time(s) at which chocolate cravings tend to occur.” and “If YES, please explain what tends to trigger your chocolate cravings.”). Questions about perceived patterns and triggers of cravings preceded other questions related to menstrual craving to avoid priming effects. Participants referencing menstruation as the perceived cause of temporal patterns or direct trigger of cravings in their narrative responses (e.g., “menstruation,” “menstrual cycle,” “hormones,” “that time of the month,” etc.) were categorized as “menstrual cravers” based on consensus from both authors involved in coding the data.

#### Other food craving

Participants completed the Food Craving Inventory (FCI), a well-validated and widely used measure of specific food cravings [22]. The FCI quantifies the “frequency” of cravings for 28 foods, along with the frequency of “giving in” to those cravings. Ratings load on to four factors, quantifying cravings for “high fat foods” (Cronbach’s α = .76 for “frequency, .83 for “giving in”), “sweets” (Cronbach’s α = .86, .87), “carbohydrates/ starches” (Cronbach’s α = .80, .82), and “fast food fats” (Cronbach’s α = .80, .81).

#### Acculturation

“Second generation immigrant” and “foreign-born” women completed the 32-item Stephenson Multigroup Acculturation Scale (SMAS). We calculated indices of “dominant” (DSI, 15 items, Cronbach’s α = .92, e.g., “I feel at home in the United States.”) versus “ethnic society immersion” (ESI, 17 items, Cronbach’s α = .80, e.g., “I like to listen to music of my ethnic group.”) by averaging scores on SMAS items quantifying familiarity and comfort with U.S. versus native language, culture, and cuisine [23] (please see S1 Appendix for information on how SMAS subscale scores were calculated). Respondents also completed the 12-item “U.S.” and “native cultural competency” subscales of the Abbreviated Multidimensional Acculturation Scale (AMAS, Cronbach’s *α* = .90, .89, respectively), a measure of cultural identity, language, and competence [24] (e.g., “How well do you know popular American television shows?” and “How well do you know political leaders from your native culture?”).

#### Statistical analyses

All data analyses were conducted using SPSS version 24. Men (*n* = 208) and native English speakers raised abroad (*n* = 52) were excluded from the analyses presented here. The remaining respondents were categorized as “American” (*n* = 101, 36.7%, born in the U.S., native English speaker), “second generation immigrant” (*n* = 93, 33.8%, born in the U.S., grew up speaking foreign language or bilingual), or “foreign-born” (*n* = 81, 29.5%, born outside the U.S., native language is not English). Please note that we use the term “American” to describe native English speakers with U.S.-born parents, but acknowledge that respondents categorized as “second generation immigrant” or “foreign born” may also identify as “American.” Groups (i.e., “American,” “second-generation immigrant,” and “foreign-born”) were compared in a series of chi-square (for “yes/no” questions regarding the presence of cravings) and uni- and multivariate (for those measures containing multiple subscales) analyses of variance (ANOVAs), with “group” as the fixed factor and continuous ratings (i.e., age, BMI, years lived in the U.S., and FCI, SMAS and AMAS subscale scores) as the dependent variables.

A priori power analyses indicated minimum sample sizes of 108 and 164 for adequate power (≥.80) to detect medium-sized effects in chi-square tests and multivariate analyses of variance [25]. Due to missing data, the number of respondents included in each analysis ranged from 145 to 173 for analyses including “second-generation immigrant” and “foreign-born” women, and from 99 to 271 for analyses including all respondents. Unless otherwise noted, multivariate main effects are reported in the text; descriptives and results from univariate ANOVAs and MANOVA between-subjects effects are presented in Tables.

## Results and discussion

Categorization of respondents as “American,” “second-generation immigrant,” and “foreign-born” based on birthplace and native language was supported by significant multivariate main effects of “group” on SMAS [*F* (2, 142) = 36.85, Wilk’s λ = .66, *p* < .001, $\eta_{p}^{2}$ = .34] and AMAS scores [*F* (2, 166) = 50.10, Wilk’s λ = .62, *p* < .001, $\eta_{p}^{2}$ = .38], with “second-generation immigrant” women scoring significantly higher on the SMAS DSI and AMAS “U.S. cultural competency” subscales, and significantly lower on the SMAS ESI and AMAS “native cultural competency” subscales, compared to “foreign-born” women (see Table 1 for descriptives and tests of between-subjects effects). “Foreign-born” women reported significantly fewer years of living in the U.S.; groups did not differ in age or body mass index (see Table 1 for descriptives and results from univariate ANOVAs).

**Table 1:** Demographics and acculturation in “American,” “Second Generation Immigrant,” and “Foreign-Born” respondents.

	American (*n* = 101)	Second Generation (*n* = 93)	Foreign-Born (*n* = 81)	
	*M* (*SD*)	*M* (*SD*)	*M* (*SD*)	Statistic
Demographics				
Age	19.57 (3.63)	19.00 (1.11)	19.90 (1.69)	*F* (2, 268) = 2.90, *p* = .06, *η*^2^ = .02
Body Mass Index	24.07 (4.03)	24.45 (6.37)	23.39 (9.39)	*F* (2, 250) = .50, *p* = .61, *η*^2^ = .004
Years lived in U.S.	n/a	18.12 (3.35)	5.94 (6.10)	*F* (1, 171) = 273.21, p < .001, *η*^2^ = .62
Stephenson Multigroup Acculturation Scale				
Dominant Society Immersion	n/a	3.60 (.30)	2.89 (.67)	*F*(1,143) = 67.72, *p* < .001, *η*^2^ = .32, 95% C.I.: .54, .89
Ethnic Society Immersion	n/a	3.06 (.40)	3.33 (.44)	*F*(1,143) = 15.13, *p* < .001, *η*^2^ = .10, 95% C.I.: -.41, -.13
Abbreviated Multicultural Acculturation Scale				
U.S. Cultural Competency	n/a	3.32 (.51)	2.67 (.58)	*F*(1,167) = 59.51, *p* < .001, $\eta_{p}^{2}$ = .26, 95% C.I.: .48, .81
Native Cultural Competency	n/a	2.32 (.63)	2.98 (.68)	*F*(1,167) = 42.66, *p* < .001, $\eta_{p}^{2}$ = .20, 95% C.I.: -.86, -.46

“Foreign-born” women represented a wide range of countries and cultures (Table 2). “Second-generation immigrant” women reported speaking the following native languages growing up: Spanish (*n* = 31), English (exclusively, or in addition to a non-English native language, *n* = 18), Chinese (*n* = 7, Wenzhounese, Fuzhounese, Cantonese, or Mandarin), French or Haitian Creole (*n* = 6), French (*n* = 4), Bengali, Farsi, Korean, Malayalam, Polish, Russian, and Twi (*n* = 2 each), Bambara, German, Gujarati, Hebrew, Igbo, Italian, Patwa/ Patois, Punjabi, Urdu, and Vietnamese (*n* = 1 each). “Foreign-born” women represented the following native languages: Chinese (Cantonese or Mandarin, *n* = 30), Korean (*n* = 10), Spanish (*n* = 9), French or Haitian Creole and Japanese (*n* = 5 each), Hindi (*n* = 3), English (exclusively, or in addition to a non-English native language, *n* = 3), German and Twi/ Akan(*n* = 2 each), Arabic, Bangla/ Bengali, Bemba, Hungarian, Krio, Maori, Patois, Polish, Portuguese, Swedish, Tagalog, Triqui/ Trique and Turkish (*n* = 1 each).

**Table 2:** Home continents and countries of foreign-born respondents.

Continent	Country
Africa (*n* = 5)	Ghana (*n* = 2), Sierra Leone, Sudan and Zambia (*n* = 1 each)
Asia (*n* = 49)	China (*n* = 25), South Korea (*n* = 10), Japan (*n* = 5), India (*n* = 3), Hong Kong and Taiwan (*n* = 2 each), Bangladesh and Philippines (*n* = 1 each),
Europe (*n* = 6)	Germany (*n* = 2), Hungary, Ukraine, Poland and Sweden (*n* = 1 each)
South America (*n* = 5)	Ecuador (*n* = 2), Brazil, Peru and Venezuela (*n* = 1 each)
North America (*n* = 14)	Dominican Republic and Haiti (*n* = 5 each), Puerto Rico (*n* = 2), Mexico and Jamaica (*n* = 1 each)
Other (*n* = 2)	New Zealand (*n* = 1, native Maori speaker), Turkey (*n* = 1)

There were no significant multivariate main effects of “group” on combined FCI “frequency” [*F* (8, 482) = .98, Wilk’s λ = .97, *p* = .45, $\eta_{p}^{2}$ = .02] or “giving in” subscale scores [*F* (8, 460) = 1.13, Wilk’s λ = .96, *p* = .34, $\eta_{p}^{2}$ = .02]. “Foreign-born” women were significantly less likely to have experienced any chocolate craving (Table 3), compared to “American” (post-hoc *p* = .02) and “second generation immigrant” women (post-hoc *p* = .03). There were no between-group differences in prevalence of “regular” chocolate cravings (Table 3).

**Table 3:** Chocolate cravings in “American,” “Second Generation Immigrant,” and “Foreign-Born” respondents.

	American (*n* = 101)	Second Generation (*n* = 93)	Foreign-Born (*n* = 81)	Statistic
	% (*n*)	% (*n*)	% (*n*)	
Ever craved chocolate	91.9 (91)	92.1 (82)	80.0 (64)	*χ*^2^ = 7.93, *p* = .02, *φ* = .17
Regular chocolate craving	36.0 (36)	34.8 (31)	40.0 (32)	*χ*^2^ = .53, p = .77, *φ* = .04
Menstrual chocolate craving	32.7 (33)	40.9 (38)	17.3 (14)	*χ*^2^ = 11.50, *p* = .003, *φ* = .21

A third of all respondents endorsed experiencing “menstrual” chocolate craving (30.9%, *n* = 85), meaning that they perceived their chocolate cravings to coincide with a specific time of the menstrual cycle or identified menstruation, premenstrual symptoms, ovulation, or other aspects of the menstrual cycle as a direct trigger of chocolate cravings. “Foreign-born” women were significantly less likely to link chocolate cravings to the menstrual cycle, compared to “American” (post-hoc *p* = .03) and “second generation immigrant” women (post-hoc *p* = .002; Table 3). Respondents craving chocolate non-menstrually indicated observing the following temporal patterns or triggers of their chocolate craving episodes (overlapping responses, i.e., respondents often indicated more than one pattern or trigger): when in a specific mood state (*n* = 17, e.g., “down,” “stressed,” “blue” or “upset”), at a specific time of day (*n* = 12, predominantly in the afternoons or evenings), when studying (*n* = 8), during certain holidays (*n* = 4, specifically Halloween, Valentine’s day, Easter, and Christmas), following meals (*n* = 3), when thinking about chocolate (*n* = 2), while on vacation (*n* = 1), and when wanting to “satisfy my sweet tooth” (n = 1).

In “second generation immigrant” and “foreign-born” respondents there were significant multivariate main effects of “menstrual craving” on combined SMAS [*F* (2, 142) = 11.58, Wilk’s *λ* = .86, *p* < .001, $\eta_{p}^{2}$ = .14] and AMAS subscale scores [*F* (2, 166) = 10.00, Wilk’s *λ* = .89, *p* < .001, $\eta_{p}^{2}$ = .11], with menstrual cravers scoring significantly higher on the DSI [*M* = 3.59, *SD* = .38 vs. *M* = 3.08, *SD* = .66; *F* (1, 143) = 23.26, *p* < .001, $\eta_{p}^{2}$ = .14, 95% C.I.: -.72, -.30] and “U.S. cultural competency” subscales [*M* = 3.32, *SD* = .52 vs. *M* = 2.89, *SD* = .63; *F* (1, 167) = 17.87, *p* < .001, $\eta_{p}^{2}$ = .10, 95% C.I.: -.63, -.23], and significantly lower on “native cultural competency” [*M* = 2.43, *SD* = .66 vs. *M* = 2.71, *SD* = .75; *F* (1, 167) = 5.36, *p* = .02, $\eta_{p}^{2}$ = .03, 95% C.I.: .04, .52].

Taken together, our data thus suggest no significant differences between the three groups in the prevalence of non-chocolate food cravings or “regular” cravings for chocolate. Menstrual craving, on the other hand, was least prevalent in “foreign-born” women, and associated with greater levels of U.S. acculturation and lower native cultural competency. Results thus provide compelling support for the view of menstrual chocolate craving as a potentially culture-bound syndrome. Findings support our hypothesis that exposure to U.S. culture and the “thin ideal” of female beauty create the perception that desires for seemingly “taboo” foods like chocolate must be justified with socially acceptable excuses like menstruation or premenstrual syndrome (PMS). It has been speculated that similar cultural norms about craving and food intake in pregnancy may put American women at especially high risk for excess gestational weight gain [26].

Our findings are consistent with prior research suggesting that premenstrual syndrome or “PMS” is socially constructed [27] and Western culture-specific [28]. Recent research also finds that risk of premenstrual dysphoric disorder (PMDD) in ethnic minority women in the U.S. is a direct function of exposure to American culture and increases with increasing amount of time spent residing in the U.S. [29]. Interestingly, our measures did not specifically quantify dietary acculturation. The perceived association between the menstrual cycle and chocolate craving thus appears to be transmitted largely implicitly, via broad exposure to U.S. media, history, and politics. More research is needed to examine the hypothesis that menstrual craving is the product of internalized norms about standards of female beauty, “forbidden” foods, and socially sanctioned cues that signal permission to indulge.

There are several limitations to the research that must be noted. First, “second-generation” and “foreign-born” respondents were not a homogeneous group, but represented individuals from various cultures. Given our focus on examining differences between groups with varying levels of U.S. acculturation, collapsing these respondents into two groups is justifiable; however, future research should examine the extent to which findings may differ if looking separately at individuals from different regions of the world with varying degrees of cultural similarities and differences, compared to the U.S. and North America. We did not collect information on frequency of consumption or liking of chocolate. Women also did not indicate information on regularity of their menstrual cycle or use of hormonal or other forms of contraception. Given that menstrual chocolate craving has been shown to be unrelated to levels of ovarian hormones involved in regulating the menstrual cycle, these factors are however relatively unlikely to have influenced our findings [19,21,30].

Another important limitation is our focus on one very specific example of craving, namely chocolate craving and its perceived association with the menstrual cycle. We focused on this example because the phenomenon of menstrual chocolate craving has been explored somewhat more thoroughly in the published literature, compared to other types and targets of craving, in particular in regards to the role of cultural context in craving etiology [16,18–20,31]. Of note, there is evidence to suggest that a perceived link between menstruation and craving in women may extend to other substances. For example, research on nicotine addiction suggests a possible effect of hormonal fluctuations on abstinence-related symptoms, with studies finding evidence for a heightened experience of withdrawal and increased cue-induced craving during the luteal and ovulatory phases of the menstrual cycle [32,33]. Similarly, alcohol-dependent women report increased frequency of drinking during the premenstrum, and they specifically identify this time of the menstrual cycle as a drinking cue [34]. More research is needed to explore the extent to which findings presented here generalize to our understanding of cravings in other domains.

## Conclusions

In sum, we present evidence for a role of acculturation in the experience of menstrual chocolate craving. In the absence of significant between-group differences in overall chocolate and other food craving prevalence, these results suggest that exposure to U.S. culture increases the likelihood that menstruation is identified as a perceived trigger or cause of chocolate cravings, perhaps in an effort to justify consumption of an otherwise “forbidden” food. Findings have several important implications. They inform our understanding of diet-related health in immigrants to the U.S., who are initially at relatively lower risk of overweight (the “healthy migrant effect”) [35], an advantage that dissipates as a function of decreased consumption of ethnic foods [36]. Menstrual craving has been linked to higher body mass and elevated eating disorder symptomology [37], suggesting that it may be another potentially preventable negative consequence of dietary acculturation specifically in women.

Importantly, our findings also add to growing evidence suggesting that the craving construct, or at least certain elements of the craving experience, may be uniquely meaningful in North America. For example, most languages outside of English lack a fully equivalent translation of the term “craving” [38]. It is increasingly recognized that sociocultural factors can dramatically alter the course and outcomes of disorders traditionally attributed solely to biological abnormalities (e.g., schizophrenia) [39]. Future research should examine the impact of acculturation on the experience and manifestation of conditions characterized by recurrent cravings, including addictions, eating and weight disorders, paraphilias, and impulse control and obsessive-compulsive disorders.

## Supporting information

S1 Appendix Study measures. Chocolate craving questions and questions and scoring for the Stephenson Multigroup Acculturation Scale.
(DOCX)
