# Supplementary material for: Does culture create craving? Evidence from the case of menstrual chocolate craving
Source: PLoS One. 2017 Jul 19;12(7):e0181445. doi: 10.1371/journal.pone.0181445 (PMC5517000; doi:10.1371/journal.pone.0181445)
Supplement: S1 Appendix — Chocolate craving questions and questions and scoring for the Stephenson Multigroup Acculturation Scale. (DOCX) [file pone.0181445.s001.docx]

**Chocolate Craving Questions**

1. Have you ever experienced a craving for chocolate? Yes No
2. Do you crave chocolate regularly? Yes No
3. If you experience any chocolate cravings, do you feel that they occur at any specific point in time (i.e. time of year, month, day, etc.)? Yes No
4. If YES, please specify the time(s) at which chocolate cravings tend to occur.
5. If you experience any chocolate cravings, do you feel that they are caused by any particular triggers? Yes No
6. If YES, please explain what tends to trigger your chocolate cravings.

**Stephenson Multigroup Acculturation Scale**

ESI = “ethnic society immersion”

DSI = “dominant society immersion”

[R] = item was reverse scored

1. I understand English, but I'm not fluent in English. [R] **DSI**
2. I am informed about current affairs in the United States. **DSI**
3. I speak my native language with my friends and acquaintances from my country of origin. **ESI**
4. I have never learned to speak the language of my native country. [R] **ESI**
5. I feel totally comfortable with (Anglo) American people. **DSI**
6. I eat traditional foods from my native culture. **ESI**
7. I have many (Anglo) American acquaintances. **DSI**
8. I feel comfortable speaking my native language. **ESI**
9. I am informed about current affairs in my native country. **ESI**
10. I know how to read and write in my native language. **ESI**
11. I feel at home in the United States. **DSI**
12. I attend social functions with people from my native country. **ESI**
13. I feel accepted by (Anglo) Americans. **DSI**
14. I speak my native language at home. **ESI**
15. I regularly read magazines of my ethnic group. **ESI**
16. I know how to speak my native language. **ESI**
17. I know how to prepare (Anglo) American foods. **DSI**
18. I am familiar with the history of my native country. **ESI**
19. I regularly read an American newspaper. **DSI**
20. I like to listen to music of my ethnic group. **ESI**
21. I like to speak my native language. **ESI**
22. I feel comfortable speaking English. **DSI**
23. I speak English at home. **DSI**
24. I speak my native language with my spouse or partner. **ESI**
25. When I pray, I use my native language. **ESI**
26. I attend social functions with (Anglo) American people. **DSI**
27. I think in my native language. **ESI**
28. I stay in close contact with family members and relatives in my native country. **ESI**
29. I am familiar with important people in American history. **DSI**
30. I think in English. **DSI**
31. I speak English with my spouse or partner. **DSI**
32. I like to eat American foods. **DSI**
